# The changing contribution of childhood social characteristics to mortality: a comparison of Finnish cohorts born in 1936–50 and 1961–75

**DOI:** 10.1093/ije/dyaa041

**Published:** 2020-04-04

**Authors:** Pekka Martikainen, Irma Elo, Lasse Tarkiainen, Janne Mikkonen, Mikko Myrskylä, Heta Moustgaard

**Affiliations:** d1 Population Research Unit, Faculty of Social Sciences, University of Helsinki, Helsinki, Finland; d2 Centre for Health Equity Studies (CHESS), Stockholm University and Karolinska Institutet, Stockholm, Sweden; d3 Max Planck Institute for Demographic Research, Rostock, Germany; d4 Population Studies Center, University of Pennsylvania, Philadelphia, PA, USA; d5 Department of Social Policy, London School of Economics and Political Science, London, UK

**Keywords:** Mortality, childhood, life course, cohort study, social determinants

## Abstract

**Background:**

Life course epidemiology suggests that early life circumstances affect adult mortality, but most of the evidence is based on cohorts born in the beginning of the 20th century. It remains unclear whether and how the influences of early life circumstances on mortality have changed in later birth cohorts.

**Methods:**

Analyses rely on 10% register-based samples of households drawn from the 1950 and the 1975 Finnish censuses, with consistent follow-up of socioeconomic and housing-related characteristics and early mid-life mortality (at ages 30–55 years). We estimate survival models for the associations between childhood circumstances and all-cause, internal and external mortality for cohorts born in 1936–50 and 1961–75 adjusting for attained social characteristics. We estimate sibling intraclass correlations as summary measures of all early life and familial influences.

**Results:**

Adverse childhood social circumstances were typically associated with about 10**–**30% excess cause-specific mortality. These associations were almost fully attenuated by adjustment for achieved later life social characteristics. Early life influences have grown over time for mortality from external causes, particularly as related to home ownership and family type. Differentials have remained stable for internal causes. The intraclass correlations further confirmed the increasing association of early life circumstances on external-cause mortality.

**Conclusions:**

Our analyses show that the associations between childhood characteristics and mid-life mortality are substantial and almost fully mediated by achieved adult social characteristics. The increase in the contribution of childhood circumstances to mid-life mortality is driven by ever stronger associations with external causes of death.

**Key Messages**

- Life course epidemiology suggests that early life circumstances affect adult mortality, but it remains unclear whether these influences have changed over time.
- We used two samples of Finnish households drawn from the 1950 and 1975 Finnish censuses, with identifiers for families and households making it possible to identify all family members.
- We demonstrate substantial associations between early life socioeconomic and housing-related circumstances and mid-life cause-specific mortality, which are almost fully explained by achieved social and family characteristics.
- For external-cause mortality, these associations have grown for the more recent birth cohorts, with the increase for housing-related characteristics being particularly strong.
- For internal-cause mortality, the associations of childhood characteristics have remained more stable.

## Introduction

Social inequalities in mortality have been extensively documented.^1^^,^^2^ Until fairly recently, most studies of social inequalities in adult mortality have focused on the role of adult characteristics. However, as evidence of the associations between childhood living conditions and adult health has accumulated, increased attention is now being paid to the contribution of the entire life course.^3–6^ The accumulated evidence in life course epidemiology suggests that childhood nutritional status, health, socioeconomic status, place of residence and other household characteristics predict adult health and mortality.^7–13^ The mechanisms through which childhood environment is hypothesized to influence adult health include indirect mechanisms operating through attained adult social characteristics (e.g. education, other socioeconomic status and lifestyle) and direct effects of *in utero* programming and childhood health.^14^

Most of the evidence on the contribution of childhood conditions to adult mortality is based on historical cohorts and cohorts born in the early part of the 20th century.^4^^,^^5^^,^^8^ The conditions at the time when these cohorts grew up were characterized by high rates of infectious diseases, poor nutrition and hygiene and high rates of poverty and material hardship. The findings obtained from these earlier cohorts may no longer apply to more recent cohorts. In the latter half of the 20th century, Western countries experienced rapid economic growth, social changes and improvements in health conditions and overall standard of living. However, because of the simultaneously decreasing household size, postponement of marriage and childbearing, higher rate of union dissolution and overall diversification of family forms, more recent cohorts have experienced a more diversified childhood family structure than earlier cohorts.

These changes may have also influenced the mechanisms or pathways through which childhood circumstances influence adult health and mortality. Changes in economic structure, educational expansion and changing family forms have gradually transformed the work and family lives of cohorts that entered adulthood in the second half of the 20th century. Similarly, changing patterns of lifestyle risk factors— for example, the emergence and the decline of the smoking epidemic and the arrival of an obesogenic food environment— have influenced the health of more recent cohorts. Currently, there are no studies that we know of that compare the influence of early life circumstances on adult mortality of cohorts born before and after mid-20th century. In this paper, we: (i) compare the associations between various childhood circumstances and adult total and cause-specific mortality in Finland across cohorts born in 1936–50 and 1961–75; (ii) examine whether the effects of childhood characteristics are operating through attained adult social characteristics; and (iii) examine whether these effects may have changed across the two cohorts.

## Methods

### Participants and mortality follow-up

We used two 10% random samples of households drawn from the 1950 and 1975 Finnish censuses. The samples were linked with register-based follow-up of death records using personal identifiers available to all permanent residents of Finland (Statistics Finland permission TK-53-789-10 and TK-53-155-17). We included individuals aged 0**–**14 years, living in private households at the time of the 1950 (birth cohorts 1936–50, *n* = 117 061) and the 1975 census (birth cohorts 1961–75, *n* = 102 280). The census data contained identifiers for families and households making it possible to identify family members. We used these identifiers in linking children with information on their parents’ characteristics. The 1950 sample has been linked to quinquennial census records in the years 1970–95 and to death records for the years 1970–2007.^15–18^ The 1975 sample was designed to be identical in structure; with linkage to census records in 1995, 2000 and 2005 and to death records until the end of 2016. For both samples we measured childhood characteristics from the household census (i.e. at age 0**–**14) and the individual’s own educational attainment from the first quinquennial end-of-year census after the subjects turned 30. Adult characteristics were thus measured at ages 30**–**34 depending on the birth year.

**Figure 1:**
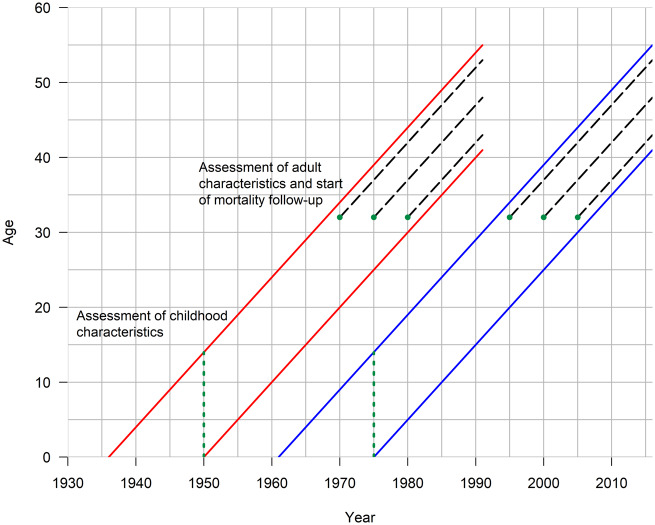
Study design and measurement for the 1950 and 1975 samples; Finnish men and women aged 30–34 at baseline.

Mortality follow-up begun at the time of the assessment of adult characteristics at age 30**–**34. Of the sampled children, we excluded those whose parents could not be identified and those who, due to death or emigration, were not in the census when adult characteristics were measured (1950: *n* = 19 184, 1975: *n* = 8331). To guarantee comparability in the mortality follow-up, we followed the 1975 cohort to the end of 2016 and the 1950 cohort to the end of 1991 until the cohort members were aged 41**–**55. Cause of death was classified according to the 8th, 9th or 10th revision of the International Classification of Diseases and Deaths (ICD) with coding made comparable across the ICD revisions by Statistics Finland. Our final analyses were based on 1 467 360 person-years and 3592 deaths in the 1950 sample and 1 527 929 person-years and 2491 deaths in the 1975 sample. The design of the study is shown in Figure 1.

### Childhood characteristics

Childhood characteristics came from the 1950 or the 1975 censuses when the cohort members were aged 0**–**14. We measure both socioeconomic and housing-related characteristics. For highest parental occupational class we distinguished the following groups: (i) non-manual, (ii) manual, (iii) farmers, (iv) employers/self-employed, and (v) others. We coded highest parental education as (i) upper secondary or higher, (ii) lower secondary and (iii) primary school or less. Of the housing-related characteristics, home ownership included: (i) home owners, (ii) renters (including those for whom the employer provided housing) and (iii) others/unknown. We characterized childhood housing conditions by household crowding coded as the number of persons per heated room in the dwelling (<2, 2.0**–**2.99, 3.0**–**3.99, 4+ and unknown). Childhood family structure distinguished: (i) two- parent and (ii) single- parent families.

### Adult characteristics

Adult characteristics were measured in subsequent censuses when the participants were aged between 30 and 34 years (the first 5-year census after turning 30) when most had completed their education and begun occupational and family careers. Occupational class was divided into (i) non-manual, (ii) manual, (iii) farmers, (iv) employers/self-employed and (v) others. Unemployed and retired persons were classified according to their previous occupations; housewives were categorized according to the occupation of the head of household. We distinguished between four educational levels: (i) higher tertiary (16+ years) education, (ii) lower tertiary (13**–**15 years), (iii) secondary (11**–**12 years) and (iv) basic or primary (9 years). Home ownership included (i) home owners, (ii) renters (including those for whom the employer provided housing) and (iii) others and unknown. Marital status was coded as (i) married, (ii) divorced/separated/widowed and (iii) never married or unknown.

### Causes of death

We examined all-cause mortality as well as alcohol=related, accidental and violent causes of death (ICD-10 F10, G312, G4051, G621, G721, I426, K292, K70, K860, K852, 0354, P043, Q860, V01-Y89 as the underlying cause) separately, as these are common among Finnish people.

### Statistical methods

We used mixed-effects survival models to estimate all-cause and cause-specific mortality from the age of 30**–**34 until the end of follow-up, which for the oldest individuals was 55 years. The baseline hazard was assumed to follow the Weibull distribution stratified by sex (we tested for sex interactions but these were negligible). As the data consist of persons nested in families, we use a two-level random intercept specification to take account of family clustering and allow for the estimation of family- specific random intercepts. The results are presented as hazard ratios based on coefficients from the proportional hazard parameterization [hazard ratio (HR) = e^β^] and their 95% confidence intervals (CIs). To test for change in the associations over the two samples, we pooled the data and included interactions with birth cohort.

In order to assess the similarity in survival between siblings, we used the accelerated failure-time metric of the same model to estimate the family- level variance θ. Because the individual level error term in Weibull models follows the Gumbel distribution, the individual- level variance is $\frac{\pi^{2}}{6p^{2}}$ where p is the shape parameter of the Weibull distribution and is obtained from the model. The sibling correlation measuring the sibling similarity in mortality (intraclass correlation is the share of total variation in mid-life mortality which was explained by between-family differences) and is calculated according the method suggested by Goldstein *et al*.^19^ for each model:

$$\mathrm{Intraclass}\;\mathrm{correlation}=\frac{\theta }{\theta +\frac{\pi^{2}}{6p^{2}}\;}$$

We take the sibling intraclass correlation to be a summary measure of all early life influences, both observed and unobserved, that are shared by siblings,^6^ thus reflecting shared genetic and social influences. The ‘mestreg’ command in Stata 14.2 was used for the estimation and ‘nlcom’ command for obtaining the 95% confidence intervals for the sibling correlation.

## Results

The later cohorts had more highly educated parents who were much more likely to be employed in professional occupations (Table 1). Similarly, they had somewhat higher levels of education and were somewhat more likely to be employed in professional occupations than the cohorts born before the middle of the 20th century.

**Table 1.** Number of participants and deaths according to background characteristics for the 1950 and 1975 samples; Finnish men and women aged 30–34 at baseline

	1950 census		1975 census	
	Sample	Deaths	Sample	Deaths
	*n* = 97 871	*n* = 3592	*n* = 93 940	*n* = 2495
Gender				
Male	52.0	74.2	51.2	69.8
Female	48.1	25.8	48.8	30.2
Region				
Western Finland	33.2	28.5	36.8	34.4
Southern Finland	32.9	32.8	35.4	35.3
Eastern Finland	19.1	22.0	13.3	14.8
Northern Finland	14.9	16.7	14.5	15.6
Childhood characteristics				
Highest parental education				
Primary or less	89.8	91.7	45.9	56.2
Lower secondary	6.0	5.2	28.1	25.1
Upper secondary or higher	4.2	3.1	26.0	18.7
Occupation of family head				
Non-manual	15.0	12.8	32.4	26.1
Manual	41.6	43.4	46.1	50.1
Farmers	34.1	34.7	11.0	11.6
Employer/self-employed	7.6	7.1	6.2	5.7
Other	1.7	2.1	4.3	6.5
People per heated room				
<2	32.2	28.2	66.4	60.7
2.0-2.99	31.9	31.2	21.7	23.8
3.0-3.99	16.4	17.5	6.3	7.8
4+	18.3	21.9	5.0	7.0
Unknown	1.1	1.3	0.6	0.7
Home ownership				
Owner	60.8	62.2	60.6	59.0
Renter	32.2	30.4	37.2	37.6
Unknown	7.0	7.4	2.2	3.3
Family type				
Two parents	91.1	88.0	90.4	86.6
Single parent	8.9	12.1	9.6	13.4
Adult characteristics				
Educational attainment				
Higher tertiary	4.8	2.6	12.0	3.9
Lower tertiary	7.5	4.7	28.4	14.6
Secondary	38.3	29.6	45.6	49.0
Basic	49.4	63.1	14.0	32.6
Occupation				
Non-manual	42.9	25.9	51.0	31.0
Manual	38.6	46.3	33.3	48.1
Farmer	6.6	7.0	2.7	3.5
Employer/self-employed	4.4	4.8	5.7	5.9
Other	7.6	16.0	7.3	11.5
Home ownership				
Owner	57.3	49.6	61.0	46.0
Renter	37.7	41.2	34.9	46.4
Other/unknown	5.1	9.2	4.1	7.6
Marital status				
Married	74.8	61.4	47.1	30.1
Separated/divorced/widowed	6.5	8.7	6.3	8.7
Never married	18.7	29.8	46.6	61.2

In the 1950 sample, less educated or manual worker parents and children living in crowded or single-parent households had higher all-cause mortality in early mid-life, whereas home ownership in childhood was unassociated with early mid-life mortality (Table 2). There was no consistent evidence that these mortality differentials would have declined for the more recent birth cohorts in the 1975 sample. Instead, some of these associations appear to have increased. In particular, increase in the strength of the associations was especially strong for housing-related circumstances, such as living in rented housing (HR = 1.03 to HR = 1.21, with a cohort interaction parameter 1.17 95% CI 1.05, 1.31) in the last column) and living in single- parent households and in households with three persons per room. However, differences in mortality by parental education and occupational class have remained stable.

**Table 2.** Hazard ratios (HR) with 95% confidence interval (CI, in parentheses) for all-cause mortality for the 1950 and 1975 samples; Finnish men and women aged 30–34 at baseline

	1950 census						1975 census						
	Baseline^a^		Adult adjusted^b^		All variables^c^		Baseline^a^		Adult adjusted^b^		All variables^c^		
	HR	95% CI	HR	95% CI	HR	95% CI	HR	95% CI	HR	95% CI	HR	95% CI	Cohort interaction^d^
Highest parental education (upper secondary or higher)													
Lower secondary	1.15	(0.91, 1.46)	0.96	(0.75, 1.22)	0.96	(0.75, 1.22)	1.22	(1.08, 1.38)	1.03	(0.91, 1.17)	1.02	(0.90, 1.16)	1.06 (0.81, 1.38)
Primary or less	1.27	(1.04, 1.53)	0.80	(0.66, 0.98)	0.79	(0.65, 0.98)	1.43	(1.29, 1.59)	1.09	(0.98, 1.21)	1.06	(0.94, 1.19)	1.13 (0.90, 1.40)
Occupation of family head (non-manual)													
Manual	1.24	(1.12, 1.38)	0.91	(0.82, 1.02)	0.90	(0.81, 1.01)	1.33	(1.21, 1.46)	1.10	(0.99, 1.21)	1.10	(0.99, 1.23)	1.08 (0.93, 1.24)
Farmers	1.10	(0.99, 1.23)	0.83	(0.74, 0.93)	0.84	(0.74, 0.95)	1.14	(0.99, 1.31)	1.02	(0.89, 1.18)	1.05	(0.90, 1.22)	1.02 (0.85, 1.22)
Employer/self-employed	1.05	(0.89, 1.22)	0.84	(0.72, 0.98)	0.85	(0.72, 1.00)	1.03	(0.86, 1.24)	0.92	(0.76, 1.10)	0.94	(0.77, 1.13)	0.97 (0.76, 1.24)
Other	1.29	(1.00, 1.67)	0.93	(0.72, 1.20)	0.90	(0.69, 1.16)	1.74	(1.46, 2.08)	1.33	(1.11, 1.59)	1.30	(1.08, 1.57)	1.06 (0.98, 1.82)
People per heated room (<2)													
2.0-2.99	1.14	(1.04, 1.24)	0.99	(0.91, 1.08)	0.99	(0.91, 1.08)	1.21	(1.10, 1.33)	1.07	(0.97, 1.18)	1.06	(0.96, 1.17)	1.06 (0.93, 1.21)
3.0-3.99	1.21	(1.10, 1.35)	0.98	(0.88, 1.08)	0.97	(0.87, 1.08)	1.48	(1.27, 1.72)	1.23	(1.05, 1.43)	1.22	(1.04, 1.42)	1.22 (1.01, 1.47)
4+	1.39	(1.26, 1.53)	1.03	(0.93, 1.13)	1.01	(0.92, 1.12)	1.53	(1.30, 1.79)	1.21	(1.03, 1.42)	1.20	(1.02, 1.41)	1.12 (0.93, 1.35)
Unknown	1.47	(1.08, 2.00)	1.19	(0.87, 1.61)	1.08	(0.78, 1.49)	1.24	(0.76, 2.02)	1.11	(0.68, 1.81)	1.04	(0.64, 1.71)	0.86 (0.48, 1.52)
Home ownership (owner)													
Renter	1.03	(0.96, 1.11)	1.10	(1.02, 1.19)	1.06	(0.97, 1.15)	1.21	(1.12, 1.32)	1.05	(0.97, 1.14)	1.02	(0.94, 1.12)	1.17 (1.05, 1.31)
Unknown	1.09	(0.96, 1.24)	1.06	(0.93, 1.21)	1.03	(0.90, 1.19)	1.61	(1.29, 2.03)	1.43	(1.14, 1.79)	1.38	(1.09, 1.74)	1.46 (1.13, 1.90)
Family type (two parents)													
Single parent	1.12	(1.01, 1.24)	1.06	(0.96, 1.18)	1.05	(0.94, 1.16)	1.32	(1.17, 1.48)	1.10	(0.98, 1.24)	1.07	(0.95, 1.21)	1.17 (1.00, 1.37)

a Childhood characteristics in separate models, adjusted for age, sex and region.

b Baseline model + adult characteristics (education, socioeconomic position, home ownership and marital status).

c All childhood characteristics + adult characteristics (education, socioeconomic position, home ownership and marital status).

d Separately for each index category, tests whether the hazard ratios are equal between the two cohorts in the baseline model.

Tables 3 and 4 replicate the analyses for two broad causes of death; internal causes (Table 3) and external (alcohol- related, accidental and violent) causes of death (Table 4). For internal causes in the 1950 sample, most childhood characteristics were associated with elevated mortality, and these differentials were mostly similar to those observed in the 1975 sample with the exception of living in a household with over three people per heated room. However, the increase in the all-cause mortality differentials by childhood housing-related circumstances is mainly driven by external causes. For these causes of death, most childhood characteristics were more strongly associated with the risk of death in the 1975 than the 1950 sample. For example, the hazard ratio for single parenthood had increased by 30%.

**Table 3.** Hazard ratios (HR) with 95% confidence interval (CI, in parentheses) for internal mortality for the 1950 and 1975 samples; Finnish men and women aged 30–34 at baseline

	1950 census						1975 census						
	Baseline^a^		Adult adjusted^b^		All variables^c^		Baseline^a^		Adult adjusted^b^		All variables^c^		
	HR	95% CI	HR	95% CI	HR	95% CI	HR	95% CI	HR	95% CI	HR	95% CI	Cohort interaction^d^
Highest parental education (upper secondary or higher)													
Lower secondary	1.25	(0.88, 1.76)	1.07	(0.76, 1.51)	1.07	(0.75, 1.51)	1.28	(1.07, 1.52)	1.12	(0.94, 1.34)	1.13	(0.94, 1.36)	1.02 (0.70, 1.51)
Primary or less	1.50	(1.14, 1.99)	1.01	(0.76, 1.35)	1.00	(0.74, 1.34)	1.42	(1.22, 1.66)	1.15	(0.98, 1.35)	1.14	(0.96, 1.36)	0.95 (0.69, 1.30)
Occupation of family head (non-manual)													
Manual	1.33	(1.15, 1.54)	1.01	(0.87, 1.18)	0.97	(0.83, 1.14)	1.19	(1.04, 1.37)	1.02	(0.89, 1.17)	0.97	(0.83, 1.13)	0.90 (0.74, 1.10)
Farmers	1.23	(1.06, 1.43)	0.93	(0.79, 1.09)	0.90	(0.76, 1.07)	1.28	(1.06, 1.54)	1.15	(0.94, 1.39)	1.09	(0.88, 1.34)	1.03 (0.81, 1.31)
Employer/self-employed	1.06	(0.86, 1.32)	0.87	(0.70, 1.08)	0.85	(0.68, 1.06)	0.91	(0.69, 1.19)	0.82	(0.63, 1.08)	0.80	(0.60, 1.05)	0.84 (0.60, 1.19)
Other	1.33	(0.94, 1.86)	0.99	(0.71, 1.40)	0.91	(0.64, 1.29)	1.49	(1.15, 1.94)	1.20	(0.92, 1.56)	1.10	(0.84, 1.44)	1.12 (0.73, 1.72)
People per heated room (<2)													
2.0-2.99	1.14	(1.02, 1.28)	1.03	(0.91, 1.15)	1.03	(0.91, 1.15)	1.17	(1.02, 1.34)	1.06	(0.92, 1.22)	1.05	(0.91, 1.21)	1.02 (0.85, 1.23)
3.0-3.99	1.20	(1.05, 1.38)	1.01	(0.88, 1.16)	1.01	(0.88, 1.16)	1.56	(1.26, 1.93)	1.34	(1.08, 1.66)	1.32	(1.06, 1.64)	1.30 (1.01, 1.67)
4+	1.39	(1.22, 1.58)	1.09	(0.96, 1.24)	1.09	(0.95, 1.24)	1.39	(1.10, 1.77)	1.15	(0.91, 1.46)	1.14	(0.89, 1.45)	1.01 (0.78, 1.33)
Unknown	1.68	(1.13, 2.48)	1.42	(0.96, 2.10)	1.37	(0.90, 2.07)	1.36	(0.70, 2.64)	1.23	(0.63, 2.38)	1.19	(0.61, 2.33)	0.82 (0.38, 1.77)
Home ownership (owner)													
Renter	0.98	(0.88, 1.08)	1.07	(0.96, 1.18)	1.04	(0.93, 1.16)	1.08	(0.96, 1.22)	0.97	(0.86, 1.10)	0.95	(0.84, 1.07)	1.10 (0.94, 1.29)
Unknown	1.04	(0.87, 1.24)	1.02	(0.86, 1.22)	0.97	(0.81, 1.17)	1.50	(1.09, 2.08)	1.36	(0.99, 1.89)	1.27	(0.91, 1.77)	1.44 (1.00, 2,09)
Family type (two parents)													
Single parent	1.17	(1.03, 1.34)	1.13	(0.99, 1.29)	1.13	(0.99, 1.29)	1.26	(1.06, 1.49)	1.10	(0.93, 1.30)	1.07	(0.90, 1.28)	1.07 (0.86, 1.33)

a Childhood characteristics in separate models, adjusted for age, sex and region.

b Baseline model + adult characteristics (education, socioeconomic position, home ownership and marital status).

c All childhood characteristics + adult characteristics (education, socioeconomic position, home ownership and marital status).

d Separately for each index category, tests whether the hazard ratios are equal between the two cohorts in the baseline model.

**Table 4.** Hazard ratios (HR) with 95% confidence interval (CI, in parenthesis) for external mortality for the 1950 and 1975 samples; Finnish men and women aged 30–34 at baseline

	1950 census						1975 census						
	Baseline^a^		Adult adjusted^b^		All variables^c^		Baseline^a^		Adult adjusted^b^		All variables^c^		
	HR	95% CI	HR	95% CI	HR	95% CI	HR	95% CI	HR	95% CI	HR	95% CI	Cohort interaction^d^
Highest parental education (upper secondary or higher)													
Lower secondary	1.06	(0.75, 1.49)	0.85	(0.60, 1.19)	0.84	(0.59, 1.18)	1.19	(1.00, 1.42)	0.97	(0.81, 1.15)	0.93	(0.77, 1.12)	1.12 (0.76, 1.64)
Primary or less	1.09	(0.83, 1.42)	0.64	(0.49, 0.85)	0.63	(0.47, 0.85)	1.49	(1.28, 1.74)	1.06	(0.90, 1.23)	1.00	(0.85, 1.19)	1.37 (1.01, 1.87)
Occupation of family head (non-manual)													
Manual	1.18	(1.01, 1.38)	0.83	(0.70, 0.97)	0.84	(0.71, 1.00)	1.49	(1.30, 1.71)	1.18	(1.02, 1.36)	1.25	(1.07, 1.47)	1.28 (1.03, 1.57)
Farmers	0.98	(0.83, 1.15)	0.74	(0.62, 0.88)	0.77	(0.64, 0.93)	1.03	(0.83, 1.28)	0.93	(0.75, 1.16)	1.01	(0.80, 1.28)	1.04 (0.79, 1.36)
Employer/self-employed	1.06	(0.84, 1.34)	0.83	(0.66, 1.05)	0.87	(0.68, 1.10)	1.21	(0.94, 1.57)	1.06	(0.82, 1.37)	1.13	(0.86, 1.47)	1.12 (0.80, 1.59)
Other	1.24	(0.84, 1.82)	0.86	(0.58, 1.26)	0.86	(0.58, 1.28)	2.10	(1.65, 2.69)	1.52	(1.19, 1.94)	1.57	(1.22, 2.03)	1.68 (1.06, 2.64)
People per heated room (<2)													
2.0-2.99	1.15	(1.00, 1.31)	0.97	(0.84, 1.10)	0.96	(0.84, 1.10)	1.26	(1.09, 1.44)	1.07	(0.94, 1.23)	1.07	(0.93, 1.23)	1.09 (0.90, 1.32)
3.0-3.99	1.24	(1.06, 1.45)	0.94	(0.80, 1.10)	0.93	(0.79, 1.09)	1.44	(1.15, 1.79)	1.14	(0.92, 1.43)	1.14	(0.91, 1.43)	1.17 (0.89, 1.54)
4+	1.41	(1.22, 1.63)	0.96	(0.83, 1.12)	0.94	(0.81, 1.10)	1.72	(1.38, 2.14)	1.30	(1.04, 1.62)	1.28	(1.02, 1.60)	1.26 (0.96, 1.64)
Unknown	1.22	(0.74, 2.01)	0.92	(0.56, 1.52)	0.78	(0.46, 1.31)	1.20	(0.58, 2.44)	1.07	(0.52, 2.18)	0.95	(0.46, 1.96)	1.00 (0.42, 2.40)
Home ownership (owner)													
Renter	1.10	(0.98, 1.23)	1.14	(1.02, 1.28)	1.08	(0.95, 1.22)	1.32	(1.18, 1.49)	1.11	(0.98, 1.25)	1.08	(0.96, 1.22)	1.19 (1.01, 1.40)
Unknown	1.16	(0.96, 1.42)	1.11	(0.91, 1.36)	1.11	(0.91, 1.36)	1.77	(1.29, 2.44)	1.51	(1.10, 2.09)	1.52	(1.09, 2.10)	1.49 (1.02, 2.17)
Family type (two parents)													
Single parent	1.05	(0.89, 1.24)	0.98	(0.83, 1.16)	0.96	(0.81, 1.13)	1.38	(1.17, 1.63)	1.11	(0.94, 1.31)	1.07	(0.90, 1.27)	1.30 (1.03, 1.64)

a Childhood characteristics in separate models, adjusted for age, sex and region.

b Baseline model + adult characteristics (education, socioeconomic position, home ownership and marital status).

c All childhood characteristics + adult characteristics (education, socioeconomic position, home ownership and marital status).

d Separately for each index category, tests whether the hazard ratios are equal between the two cohorts in the baseline model.

The associations between parental characteristics and mid-life mortality were substantially attenuated after adjusting for the individual’s own adult social characteristics. This occurred for both cause of death groups and all-cause mortality. After these adjustments, the associations between childhood circumstances and mid-life mortality were modest, indicating strong mediation by achieved adult social characteristics. Further adjustment for all childhood characteristics simultaneously changed the model estimates only little.

Our estimates of sibling correlations—a summary measure of all early life and familial influences shared by siblings, both observed and unobserved—have increased for total mortality from 0.17 (95% CI 0.10, 0.24) in the 1950 cohort to 0.29 (95% CI 0.20, 0.37) in the 1975 cohort (Table 5). Sibling correlations for external causes of death increased from 0.28 (95 % CI 0.17**–**0.39) to 0.45 (95 % CI 0.36**–**0.54), and this increase cannot be explained by adjustment for achieved adult social characteristics.

**Table 5.** Sibling intraclass correlation (ICC)^a^ with 95% confidence interval (CI, in parentheses) for all-cause and cause-specific mortality for the 1950 and 1975 samples; Finnish men and women aged 30–34 at baseline

		1950 census		1975 census	
Cause of death	Model	ICC	95% CI	ICC	95% CI
All-cause mortality	Crude: age, sex, region	0.17	(0.10, 0.24)	0.29	(0.20, 0.37)
	Crude + all childhood	0.16	(0.09, 0.23)	0.27	(0.18, 0.35)
	All variables	0.15	(0.07, 0.22)	0.22	(0.12, 0.31)
Internal causes	Crude: age, sex, region	0.16	(0.04, 0.28)	0.15	(-0.08, 0.39)
	Crude + all childhood	0.15	(0.03, 0.27)	0.12	(-0.12, 0.37)
	All variables	0.14	(0.02, 0.27)	0.06	(-0.22, 0.35)
External causes	Crude: age, sex, region	0.28	(0.17, 0.39)	0.45	(0.36, 0.54)
	Crude + all childhood	0.28	(0.17, 0.39)	0.43	(0.34, 0.52)
	All variables	0.25	(0.13, 0.36)	0.40	(0.30, 0.50)

a Sibling intraclass correlation (ICC) measures the sibling similarity in mortality and is a summary measure of all early life influences.

## Discussion

We evaluated the association between childhood circumstances and mid-life mortality in two Finnish cohorts, born in 1936–50 and 1961–75. We showed persistent associations between early life socioeconomic and family circumstances and mid-life mortality which are almost fully mediated through achieved social characteristics at ages 30–34. Despite the extremely rapid modernization and increasing affluence of the Finnish society in the latter part of the 20th century, these associations have not decreased between the cohorts. On the contrary, similarly to increased mortality differentials by adult social characteristics,^20–22^ differentials according to childhood circumstances have increased over time for external causes of death. These increases have been substantial enough for housing-related childhood conditions to have a corresponding effect on all-cause mortality. For internal causes the associations of childhood characteristics have remained stable.

Most of the evidence on the association between childhood socioeconomic characteristics and later life mortality is from cohorts born in the first decades of the 20th century. Comparing separate studies that have recruited participants from several birth cohorts seems to tentatively indicate that the associations have not disappeared in more recent cohorts.^9^ However, this conclusion is based on qualitative comparison of distinct studies using different methodologies, statistical adjustments and social contexts. Using consistent approaches over birth cohorts we show that, despite consistent temporal change towards improving childhood material living conditions, the ‘long arm of childhood’ has not only remained robust but has grown stronger. This increase is particularly evident for childhood experiences of rented accommodation, single- parent household and, in the case of alcohol- related, accidental and violent causes, also with having a primary educated or manual parental background.

The observation that alcohol- related, accidental and violent causes are particularly strongly associated with early life is not new.^23^ It is likely that these associations are driven partially by early adoption of risky behaviours, in particular alcohol and other substance use, in the more disadvantaged childhood environments with both chronic and binge-drinking being responsible. Our particular contribution, however, is to show increasing associations between several measures of childhood disadvantage with these causes of death over time. The association between childhood disadvantage and adult problem drinking is not fully conclusive.^24^^,^^25^ For the purposes of this study, reliable evidence on alcohol use is difficult to obtain, mainly because of survey non-response, recollection and desirability biases and lack of comparative information on childhood disadvantage over different cohorts. However, it is tempting to speculate that alcohol and other substance use is ever more strongly associated with childhood disadvantage and underlies the increasing association with external mortality found in this study. It is possible that the long-term relaxation of alcohol control policies in Finland— affecting the younger cohorts more strongly— may have exacerbated these associations. Overall, these results imply that despite declining mortality in mid-life over time, a socioeconomically adverse start in life is in relative terms increasingly more hazardous to health.

Empirically disentangling the role of various life course models remains difficult. Our results and many previous analyses of childhood socioeconomic influences on mortality indicate that these associations can be best understood in a life course framework, in which the associations of childhood socioeconomic characteristics are strongly mediated through achieved later life social position,^5^^,^^26^ with own education being particularly relevant.^9^ Similar findings have also been obtained in studies on health outcomes other than mortality.^27–30^ The results also indicate that the associations of childhood characteristics on mid-life external mortality, which are mediated through attained social characteristics, have grown over time. This may come about because of at least two processes; first, the emergence of an ever-stronger association between the mediators and mortality. This possibility is supported by the fact that we know that the associations of achieved social characteristics with mortality have grown over time in Finland.^31–33^ Second, the rise of a stronger association between childhood and adult characteristics. This possibility is supported by complementary analyses presented in Supplementary Tables 1 –4, available as Supplementary data at *IJE* online. These show that the associations between childhood—in particular those between childhood family type and home ownership—and disadvantaged adult social characteristics have emerged or increased over time. This development has been particularly strong for those living in rented housing in childhood. More research to confirm and extend these findings in other settings, for other health outcomes and with other methodological approaches to disentangling between changes in direct and indirect effects, are needed.

Our analyses of sibling intraclass correlations further confirm the conclusion that the associations of early life— capturing shared genetic and social influences—with later life mortality has increased. The analyses further show that after adjustment for a wide range of observed childhood and achieved adult characteristics, the intraclass correlation remains mostly unchanged. Importantly, this finding indicates that the specific early life characteristics that we measure—characteristics that are typically also measured in earlier studies—do not fully capture the childhood characteristics that are important, nor do we fully understand the pathways though which attained adult characteristics mediate childhood influences on later life outcomes. Further research should make efforts to evaluate the contribution of unstudied childhood and adult social circumstances for these differentials, including hitherto poorly understood social and genetic influences.

### Methodological considerations

From a methodological point of view this paper contributes in several ways. First, information on childhood and adult characteristics was drawn from census records and thus was not subject to recall bias (common problem with retrospective data) or loss to follow-up (common problem with prospective data). Second, our relatively large samples enabled us to study both total and cause-specific mortality and evaluate the changing contribution of adult characteristics on the mortality differentials by childhood conditions over time. Third, we had access to two samples representing two birth cohorts 25 years apart with harmonized measurement and design.

The distributions of some of the childhood characteristics have changed between the two cohorts, making it challenging to compare point estimates between the samples. For example, in 1950, 90% of children had parents with primary education, whereas in 1975 this proportion was 46% (Table 1). Thus, our measures may differ across cohorts in their ability to differentiate between childhood circumstances. In the case of education, this distributional shift should have led to smaller differentials over time. Distributional change has been less or none for other social characteristics, and the results for these confirm our main conclusions. However, even if the distributions were similar across cohorts, their meaning may have changed. For example, around 10% of children lived in single-parent families in both cohorts, but in 1950 the single parents were more often widows whereas in 1975 they were divorced.

To overcome these measurement concerns we also assessed the contribution of all observed and unobserved childhood circumstances to the variation in mid-life mortality. This was done by calculating a sibling correlation (intraclass correlation) and comparing this measure across cohorts. The observed increase in sibling correlation is in line with our main result that many of the observed childhood characteristics had stronger associations with mortality in the more recent cohort.

In conclusion, regardless of rapid economic development in the latter part of the 20th century, we show that the associations of childhood socioeconomic characteristics and mid-life mortality are substantial. Whereas we show that these associations are mainly indirect, this does not undermine the importance of childhood circumstances as important early life course experiences that set individuals on a path of later life social achievement and health. We further show that the effects of childhood circumstances on mortality have grown for the more recent birth cohorts. To a large extent the increase in the contribution of childhood conditions on mid-life mortality is driven by ever stronger associations of childhood circumstances with alcohol- related, accidental and violent causes of death.

## Supplementary Data

Supplementary data are available at *IJE* online.

## Funding

This work was supported by the Academy of Finland [grant numbers 1294861, 1308247] and the WELLIFE project funded by NordForsk [project number 63540] .

## Conflict of Interest

None declared.

## Supplementary Material

dyaa041_Supplementary_Data
