# Supplementary material for: The changing contribution of childhood social characteristics to mortality: a comparison of Finnish cohorts born in 1936–50 and 1961–75
Source: Int J Epidemiol. 2020 Apr 4;49(3):896–907. doi: 10.1093/ije/dyaa041 (PMC7394958; doi:10.1093/ije/dyaa041)
Supplement: dyaa041_Supplementary_Data [file dyaa041_supplementary_data.docx]

Supplementary Table S1. Odds ratios^a^ (OR) with 95% confidence interval (CI, in parenthesis) for higher tertiary education according to childhood characteristics; Finnish men and women aged 30-34.

|  | 1950 census | | 1975 census | | Cohort interaction |
| --- | --- | --- | --- | --- | --- |
|  | OR | 95% CI | OR | 95% CI |  |
| Highest parental education (Upper secondary or higher) |  |  |  |  |  |
|  |  |  |  |  |  |
| Lower secondary | 0.35 | (0.31, 0.39) | 0.26 | (0.25, 0.28) | 0.74 (0.66, 0.85) |
| Primary or less | 0.07 | (0.07, 0.08) | 0.16 | (0.15, 0.17) | 2.12 (1.91, 2.35) |
| Occupation of family head (Non-manual) |  |  |  |  |  |
|  |  |  |  |  |  |
| Manual | 0.14 | (0.13, 0.15) | 0.24 | (0.23, 0.25) | 1.75 (1.59, 1.94) |
| Farmers | 0.14 | (0.13, 0.16) | 0.35 | (0.32, 0.38) | 2.45 (2.17, 2.77) |
| Employer/self-employed | 0.31 | (0.28, 0.35) | 0.41 | (0.37, 0.46) | 1.32 (1.12, 1.55) |
| Other | 0.33 | (0.26, 0.42) | 0.30 | (0.27, 0.34) | 0.92 (0.70, 0.19) |
| People per heated room (<2) |  |  |  |  |  |
| 2.0-2.99 | 0.40 | (0.37, 0.44) | 0.46 | (0.43, 0.49) | 1.15 (1.04, 1.27) |
| 3.0-3.99 | 0.23 | (0.20, 0.26) | 0.32 | (0.29, 0.36) | 1.40 (1.17, 1.66) |
| 4+ | 0.15 | (0.13, 0.17) | 0.23 | (0.20, 0.28) | 1.61 (1.30, 2.00) |
| Unknown | 0.41 | (0.30, 0.56) | 0.66 | (0.49, 0.90) | 1.60 (1.03, 2.46) |
| Home ownership (Owner) |  |  |  |  |  |
| Renter | 2.08 | (1.94, 2.23) | 0.72 | (0.68, 0.75) | 0.34 (0.32, 0.38) |
| Unknown | 1.18 | (1.03, 1.36) | 0.52 | (0.43, 0.63) | 0.44 (0.35, 0.56) |
| Family type (Two parents) |  |  |  |  |  |
| Single parent | 0.77 | (0.68, 0.88) | 0.54 | (0.50, 0.59) | 0.70 (0.60, 0.82) |
| ^a^Adjusted for age, sex and region | |  |  |  |  |

Supplementary Table S2. Odds ratios^a^ (OR) with 95% confidence interval (CI, in parenthesis) for non-manual occupation according to childhood characteristics; Finnish men and women aged 30-34.

|  | 1950 census | | 1975 census | | Cohort interaction^a^ |
| --- | --- | --- | --- | --- | --- |
|  | OR | 95% CI | OR | 95% CI |  |
| Highest parental education (Upper secondary or higher) |  |  |  |  |  |
|  |  |  |  |  |  |
| Lower secondary | 0.61 | (0.55, 0.68) | 0.45 | (0.43, 0.47) | 0.74 (0.66, 0.82) |
| Primary or less | 0.18 | (0.16, 0.19) | 0.33 | (0.32, 0.34) | 1.88 (1.72, 2.06) |
| Occupation of family head (Non-manual) |  |  |  |  |  |
|  |  |  |  |  |  |
| Manual | 0.26 | (0.25, 0.28) | 0.41 | (0.40, 0.43) | 1.56 (1.47, 1.65) |
| Farmers | 0.21 | (0.20, 0.22) | 0.36 | (0.35, 0.38) | 1.74 (1.63, 1.87) |
| Employer/self-employed | 0.36 | (0.34, 0.39) | 0.49 | (0.46, 0.52) | 1.34 (1.23, 1.47) |
| Other | 0.32 | (0.29, 0.36) | 0.40 | (0.37, 0.43) | 1.23 (1.07, 1.42) |
| People per heated room (<2) |  |  |  |  |  |
| 2.0-2.99 | 0.63 | (0.61, 0.66) | 0.66 | (0.63, 0.68) | 1.03 (0.98, 1.09) |
| 3.0-3.99 | 0.48 | (0.46, 0.50) | 0.56 | (0.53, 0.59) | 1.16 (1.08, 1.25) |
| 4+ | 0.37 | (0.35, 0.38) | 0.43 | (0.40, 0.46) | 1.19 (1.09, 1.29) |
| Unknown | 0.58 | (0.51, 0.66) | 0.61 | (0.51, 0.74) | 1.05 (0.84, 1.33) |
| Home ownership (Owner) |  |  |  |  |  |
| Renter | 1.72 | (1.66, 1.78) | 0.87 | (0.84, 0.89) | 0.50 (0.48, 0.53) |
| Unknown | 1.07 | (1.01, 1.14) | 0.65 | (0.59, 0.71) | 0.60 (0.54, 0.68) |
| Family type (Two parents) |  |  |  |  |  |
| Single parent | 0.87 | (0.82, 0.91) | 0.74 | (0.70, 0.77) | 0.85 (0.79, 0.91) |
| ^a^Adjusted for age, sex and region | |  |  |  |  |

Supplementary Table S3. Odds ratios^a^ (OR) with 95% confidence interval (CI, in parenthesis) for home ownership according to childhood characteristics; Finnish men and women aged 30-34.

|  | 1950 census | | 1975 census | | Cohort interaction^a^ |
| --- | --- | --- | --- | --- | --- |
|  | OR | 95% CI | OR | 95% CI |  |
| Highest parental education (Upper secondary or higher) |  |  |  |  |  |
|  |  |  |  |  |  |
| Lower secondary | 1.01 | (0.92, 1.10) | 1.02 | (0.99, 1.06) | 1.02 (0.93, 1.12) |
| Primary or less | 1.12 | (1.05, 1.20) | 0.91 | (0.88, 0.94) | 0.81 (0.75, 0.87) |
| Occupation of family head (Non-manual) |  |  |  |  |  |
|  |  |  |  |  |  |
| Manual | 0.99 | (0.96, 1.04) | 0.94 | (0.91, 0.97) | 0.94 (0.89, 0.99) |
| Farmers | 1.56 | (1.50, 1.63) | 1.63 | (1.55, 1.72) | 1.04 (0.98, 1.11) |
| Employer/self-employed | 1.14 | (1.07, 1.20) | 1.26 | (1.18, 1.34) | 1.11 ( 1.02, 1.21) |
| Other | 0.95 | (0.85, 1.06) | 0.75 | (0.70, 0.80) | 1.88 (0.69, 0.90) |
| People per heated room (<2) |  |  |  |  |  |
| 2.0-2.99 | 0.85 | (0.82, 0.88) | 0.84 | (0.81, 0.87) | 0.99 (0.94, 1.04) |
| 3.0-3.99 | 0.79 | (0.76, 0.82) | 0.80 | (0.76, 0.85) | 1.02 (0.95, 1.09) |
| 4+ | 0.72 | (0.69, 0.74) | 0.75 | (0.70, 0.80) | 1.05 (0.97, 1.13) |
| Unknown | 0.74 | (0.65, 0.83) | 0.99 | (0.83, 1.19) | 1.35 (1.08, 1.68) |
| Home ownership (Owner) |  |  |  |  |  |
| Renter | 0.64 | (0.62, 0.66) | 0.56 | (0.55, 0.58) | 0.88 (0.84, 0.91) |
| Unknown | 0.85 | (0.81, 0.90) | 0.85 | (0.77, 0.93) | 0.99 (0.89, 1.11) |
| Family type (Two parents) |  |  |  |  |  |
| Single parent | 0.83 | (0.79, 0.87) | 0.62 | (0.59, 0.65) | 0.75 (0.70, 0.80) |
| ^a^Adjusted for age, sex and region | |  |  |  |  |

Supplementary Table S4. Odds ratios^a^ (OR) with 95% confidence interval (CI, in parenthesis) for being married according to childhood characteristics; Finnish men and women aged 30-34.

|  | 1950 census | | 1975 census | | Cohort interaction^a^ |
| --- | --- | --- | --- | --- | --- |
|  | OR | 95% CI | OR | 95% CI |  |
| Highest parental education (Upper secondary or higher) |  |  |  |  |  |
|  |  |  |  |  |  |
| Lower secondary | 1.02 | (0.92, 1.12) | 0.87 | (0.84, 0.90) | 0.86 (0.77, 0.95) |
| Primary or less | 0.94 | (0.87, 1.01) | 0.77 | (0.74, 0.79) | 0.82 (0.75, 0.89) |
| Occupation of family head (Non-manual) |  |  |  |  |  |
|  |  |  |  |  |  |
| Manual | 0.92 | (0.88, 0.96) | 0.82 | (0.79, 0.84) | 0.89 (0.84, 0.94) |
| Farmers | 0.89 | (0.85, 0.93) | 0.89 | (0.85, 0.93) | 1.00 (0.93, 1.07) |
| Employer/self-employed | 0.98 | (0.92, 1.05) | 0.91 | (0.85, 0.96) | 0.92 (0.84, 1.01) |
| Other | 0.76 | (0.68, 0.86) | 0.76 | (0.70, 0.81) | 0.99 (0.86, 1.14) |
| People per heated room (<2) |  |  |  |  |  |
| 2.0-2.99 | 0.97 | (0.93, 1.01) | 0.91 | (0.88, 0.94) | 0.94 (0.89, 0.99) |
| 3.0-3.99 | 0.92 | (0.88, 0.97) | 0.87 | (0.82, 0.92) | 0.94 (0.87, 1.02) |
| 4+ | 0.85 | (0.81, 0.89) | 0.75 | (0.70, 0.80) | 0.88 (0.81, 0.95) |
| Unknown | 0.86 | (0.75, 0.98) | 1.07 | (0.89, 1.29) | 1.25 (0.99, 1.57) |
| Home ownership (Owner) |  |  |  |  |  |
| Renter | 0.98 | (0.95, 1.02) | 0.87 | (0.84, 0.89) | 0.88 (0.84, 0.92) |
| Unknown | 0.91 | (0.86, 0.97) | 0.95 | (0.86, 1.04) | 1.04 (0.93, 1.17) |
| Family type (Two parents) |  |  |  |  |  |
| Single parent | 0.86 | (0.81, 0.90) | 0.70 | (0.67, 0.74) | 0.82 (0.76, 0.88) |
| ^a^Adjusted for age, sex and region | |  |  |  |  |
